# Estimating the phenological dynamics of irrigated rice leaf area index using the combination of PROSAIL and Gaussian Process Regression

**DOI:** 10.1016/j.jag.2021.102454

**Published:** 2021-07-24

**Authors:** Oluseun Adeluyi, Angela Harris, Jochem Verrelst, Timothy Foster, Gareth D. Claya

**Affiliations:** a Department of Geography, School of Environment, Education and Development (SEED), University of Manchester, Manchester, United Kingdom; b Image Processing Laboratory (IPL), Parc Científic, Universitat de València, 46980 Paterna, Valéncia, Spain; c Department of Mechanical, Aerospace & Civil Engineering, University of Manchester, Manchester, United Kingdom; d Department of Strategic Space Applications, National Space Research and Development Agency, (NASRDA), Abuja, Nigeria

**Keywords:** Leaf area index, Sentinel-2, Gaussian Process Regression, Rice, Phenology, Radiative transfer model

## Abstract

The growth of rice is a sequence of three different phenological phases. This sequence of change in rice phenology implies that the condition of the plant during the vegetative phase relates directly to the health of leaves functioning during the reproductive and ripening phases. As such, accurate monitoring is important towards understanding rice growth dynamics. Leaf Area Index (LAI) is an important indicator of rice yields and the availability of this information during key phenological phases can support more informed farming decisions. Satellite remote sensing has been adopted as a proxy to field measurements of LAI and with the launch of freely available high resolution Satellite images such as Sentinel-2, it is imperative that accurate retrieval methods are adopted towards monitoring LAI at irrigated rice fields. Here, we evaluate the potential of a hybrid radiative transfer model (i.e., PROSAIL - Gaussian Process Regression (GPR), for estimating the phenological dynamics of irrigated rice LAI using imager derived from the Sentinel-2 multispectral instrument. LAI field measurements were obtained from an experimental rice field in Nasarawa state, Nigeria during the dry season. We used the PROSAIL radiative transfer model to create a look up table (LUT) that was subsequently used to train a GPR model. Afterwards, we evaluated the potential of the hybrid modelling approach by assessing the overall model accuracy and the extent to which LAI was able to accurately predict LAI during key rice phenological phases. We compared the predicted hybrid GPR LAI values with LAI values generated from the SNAP toolbox, based on a hybrid Artificial Neural Network (ANN) modelling approach. Our results show that the overall predictive accuracy of the hybrid GPR model (R2 = 0.82, RMSE = 1.65) was more accurate than that of the hybrid ANN model (R2 = 0.66, RMSE = 3.89) for retrieving LAI values from Sentinel-2 imagery. Both models underestimated LAI values during the reproductive and ripening phases . However, the accuracy during the phenological phases were more significant when using the hybrid GPR model (P < 0.05). During the different phenological phases, the hybrid GPR model predicted LAI more accurately during the reproductive (R^2^ = 0.7) and ripening (R^2^ = 0.59) phases compared to the hybrid ANN reproductive and ripening phases. When monitoring LAI phenological profiles of both hybrid models, the hybrid GPR and ANN models underestimated LAI during the reproductive and ripening phases. However, the ANN model underestimations were statistically significantly greater than those for the hybrid GPR model (P < 0.05). Our results highlight the potential of hybrid GPR models for estimating the phenological dynamics of irrigated rice LAI from Sentinel-2 data. They provided more accurate estimation of LAI patterns from varying nitrogen and water applications than hybrid ANN models.

## 1 Introduction

Grain crops are the main source of nutrition and food for populations around the world, with rice accounting for over 40% of consumption globally (Muthayya et al., 2014). Sub-Saharan Africa has one of the fastest rates of increase in rice consumption, with Nigeria accounting for 23% of the total consumption in the region (O’Donoghue and Hansen, 2017). However, sub-Saharan countries are reliant on expensive rice imports due to chronically low national yields (FAO, 2018; von Grebme et al., 2013) leading to huge financial burdens on these countries.

A wide range of factors have been proposed as causes of the large rice yield gaps observed in Nigeria and the wider sub-Saharan Africa region, including inadequate use of, and access to, inputs (e.g. water, nutrients, pest and diseases) (Fahad et al., 2017; Wahid and Close, 2007), limited farm mechanization, and a lack of expertise amongst smallholder farmers about best agronomic management practices (Hengsdijk and Langeveld, 2009). Limited access to water for irrigation and fertilizers (including nitrogen), in particular, are key factors limiting productivity and resilience of rice production in sub-Saharan Africa and other smallholder farming regions globally (Ju et al., 2009; Wang et al., 2016). Consequently, there is a growing need to monitor rice yields to address productivity gaps, including those caused by water stress and fertility limitations. Crop phenological phases inform how farm managers make decisions about application schedules (Sakamoto et al., 2005). Effective monitoring of the growth dynamics of rice crops at different phenolog-ical phases is required to help yield prediction by informing farmers as to when management interventions are necessary (Fageria, 2007). Consequently, regular monitoring of crop phenology is an important step towards improving crop productivity (Mercier et al., 2020).

Satellite remote sensing has been proposed as a potential low-cost and scalable tool for monitoring and mapping of crop yields (Gilardelli et al., 2019; Kang and Özdoğan, 2019), growth status (Pipia et al., 2019; Thorp et al., 2012; Xie et al., 2019, 2018) and stress (Bandaru et al., 2016; Banerjee et al., 2018) in agricultural environments. Key to satellite-based yield estimation approaches is the ability to accurately recover estimates of crop leaf area index (LAI) throughout the growing season. LAI is defined as half of the all-sided green leaf area per unit ground area (Chen and Black, 1991; Zheng and Moskal, 2009) and is a key biophysical parameter that reflects the physiological processes of plants, and thus is an important proxy for crop development. Satellite remote sensing represents a reliable and faster alternative to in situ LAI measurements and can detect spatiotemporally-explicit trends in LAI. The moderate resolution imaging spectroradiometer (MODIS) LAI products are often used to to estimate LA at regional to global scales (Fensholt et al., 2004; Gao et al., 2008; Jiang et al., 2010). However, the spatial resolution of MODIS (250 m) hampers its adoption for applications that require much finer spatial resolutions, such as field-level monitoring. More recently, the launch of the European Space Agency’s Sentinel-2 satellites provide a suitable platform for timely monitoring of LAI during different phenological phases due to the high spatial, spectral and temporal resolution (Drusch et al., 2012).

A broad range of methods have been developed for the retrieval of LAI from satellite imagery, which can be broadly categorized into statistical, physically-based and hybrid methods (Verrelst et al., 2015a). Statistical methods include parametric methods, such as vegetation index (VI) approaches, and non-parametric methods such as machine learning regression algorithms (MLRAs). VI-based models assume an explicit relationship between measured LAI and spectral observations in two or more bands (Clevers and Gitelson, 2012; Gitelson, 2004; Verrelst et al., 2008). The successful application of this approach has been demonstrated for a range of vegetation canopies (Darvishzadeh et al., 2008; Nguy-Robertson et al., 2012; Xie et al., 2014). Notably, VIs developed using reflectance in the red-edge region of the spectrum, such as the red-edge based NDVI and Inverted Red-Edge Chlorophyll Index (Frampton et al., 2013), have shown to estimate LAI effectively. However, VI-based developed models are often location, sensor and timespecific, making their application over large spatial extents challenging (Baret and Buis, 2008; Verrelst et al., 2015a). MLRAs have the potential to generate adaptive, robust non-linear relationships. However, they can behave unpredictably when used with spectral data exhibiting characteristics not observed during the model training phase and may tend towards over-fitting of the training dataset (Baret and Buis, 2008; Darvishzadeh et al., 2008; Rivera et al., 2014; Weiss et al., 2000). In contrast, physically-based LAI retrieval methods, which use Radiative Transfer Models (RTM) offer an explicit connection between canopy reflectance and plant biochemical and biophysical characteristics (Jacquemoud et al., 2009). The physical modelling approach takes into account the canopy architecture, illumination, soil background and viewing geometries. RTMs have frequently been applied to retrieve crop biophysical parameters from a range of different sensors (Berger et al., 2020; Darvishzadeh et al., 2008; Dorigo et al., 2007; Estévez et al., 2020). Nevertheless, the physically-based approach is not straightforward due to the trade-off between the reality and inversion possibility of the RTM made, hence, a common approach to simplify the inversion problem is by creating a Look Up Table (LUT) (Darvishzadeh et al., 2008; Weiss et al., 2000). The LUT approach simulates multiple model realizations and stores both inputs and output spectra as a LUT.

More recently, hybrid methods have emerged to circumvent some of the limitations of empirical and radiative transfer approaches. In other words, the RTM model used to create a training database of spectral reflectance and corresponding biophysical parameters and then a machine learning regression model is used to create a predictive model between the two. Consequently, hybrid methods combine the generalization level of the physically-based radiative transfer approach with the flexibility and computational efficiency of machine learning algorithms (Verrelst et al., 2019a). The Artificial Neural Network (ANN) represents the most frequently adopted MLRA used in hybrid models due to their efficient interpolation capacity. The ANN model has received much attention in biophysical variable retrieval and are currently operational as the LAI retrieval method for Sentinel-2 imagery (e.g. available within the Sentinel Application Platform (SNAP) biophysical processor toolbox)(Hu et al., 2020; Kganyago et al., 2020). However, hybrid ANN models are often difficult to train because of their multi-parameter complexity and are black box in nature (Lunagaria and Patel, 2019). Alternative approaches such as the use of hybrid Gaussian processes regression (GPR) (Rasmussen and Williams, 2006) have provided encouraging results in the framework of biophysical parameter estimation (Campos-Taberner et al., 2016; Lazaro-Gredilla et al., 2014; Verrelst et al., 2015b). For instance, Campos-Taberner et al. (2016) used hybrid GPR from simulated Sentinel-2 bands from SPOT 5 for monitoring rice crop growth patterns. Nevertheless, SPOT 5 is spectrally inferior to Sentinel-2, with no provision of the spectral band in the red-edge region, which is important for LAI estimation (Xie et al., 2019).

Therefore, the focus of the study is to evaluate the performance of two hybrid inversion approaches to derive rice LAI using Sentinel-2. - One using the more common ANN approach often applied to retrieve LAI from Sentinel-2 imagery and the other an alternative GPR approach that has shown great promise in several previous studies. Both inversion models were evaluated over the entire growing season and for their ability to predict LAI at key growth stages/phases during the growing season. Based on the properties and availability of Sentinel-2 data, this study highlights the opportunity for farmers, agronomist and researchers to use Sentinel-2 data for monitoring rice LAI in irrigated farming systems.

To achieve this, we address the following research objectives: (i) Evaluate the performance of hybrid GPR for estimating rice LAI across the entire vegetation active period and at key phenology phases of rice growth; and (ii) compare the relative performance of the hybrid GPR and hybrid ANN for estimating LAI during the different phenology phases of rice.

## 2 Data and methods

### 2.1 Study area

**Fig. 1:**
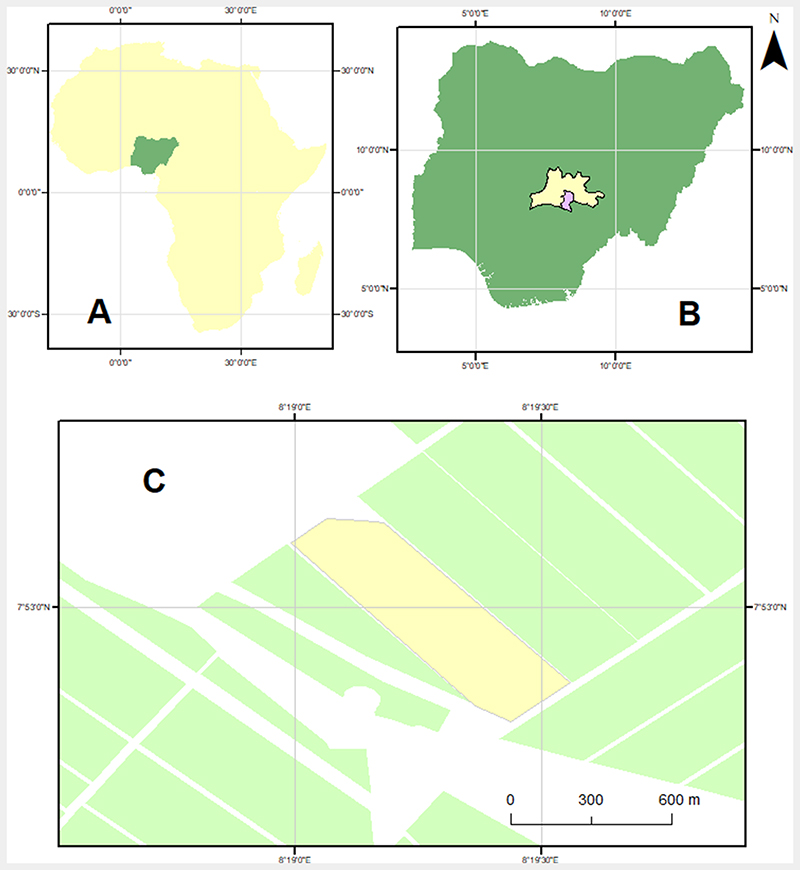
Study Area (A) Africa highlighting Nigeria in green; (B) Nigeria specifically highlighting Doma (purple) in Nasarawa state (yellow); (C) Farm showing experimental Area (yellow rectangle).

This study uses data from experimental plots located within a large rice farm (Olam farm) in the village of Rubuki about 60 km from Doma in Nasarawa State, in the North-central region of Nigeria (Fig. 1). Lowland rice is the major agricultural crop in the region, which is one of the main grain producing regions in Nigeria. The study area has a tropical humid climate with two distinct seasons: the wet (rainy) season lasts from the end of March to October, while the dry season is experienced between November and February. Maximum temperatures can reach 39 °C (March), while minimum temperatures can drop to as low as 17 °C (December/January).

### 2.2 Experimental design

**Fig. 2:**
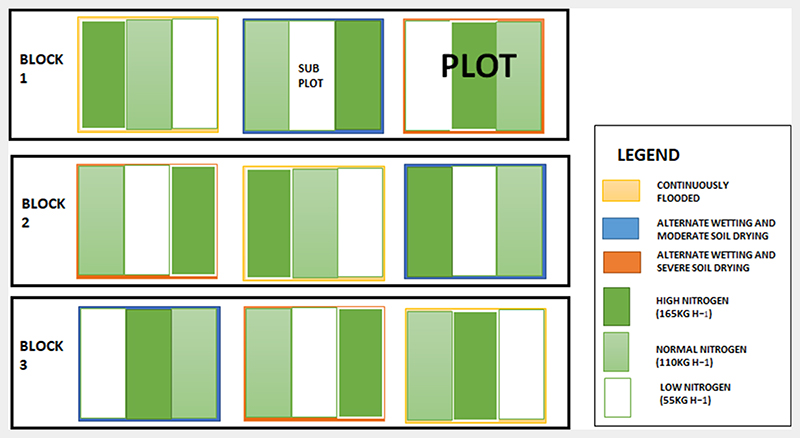
Experimental set-up for the field data collection. The experimental site was divided into three blocks. Each block was divided into three plots with each plot having three sub-plots. The treatments for each plot were divided into: continuous flooding (yellow border); alternative wetting and moderate drying (blue border); and alternative wetting and severe drying (orange border). The nitrogen applications were classed as high nitrogen (165 kg ha^−1^, dark green), normal nitrogen (110 kg ha^−1^, light green) and low nitrogen (55 kg ha^−1^, no colour).

Rice was cultivated within 27 experimental plots from December 2017 to April 2018, following a randomized split-plot design where levels of irrigation and nitrogen fertilization were varied to generate spatial and temporal variability in crop chlorophyll content and leaf area index (LAI), which is a key requirement for comparing alternative LAI retrieval approaches. The fully factorial design consisted of three irrigation regimes and three nitrogen (N) application rates. Treatments were arranged in three blocks (replications), with three plots each with three sub-plots situated within each block (3 plots × 3 sub-plots × 3 blocks = 27 sampling plots) (Fig. 2). Each plot was 77 m long and 30 m wide separated by a 2 m wide alley. Each subplot was 30 m long and 25 m wide separated by a 1 m alley (Fig. 2).

### 2.3 Irrigation and nitrogen regimes application across the phenological phases on experimental plots

Irrigation treatments consisted of three irrigation regimes, which were: (1) alternate wetting and moderate soil drying (AWMD); (2) alternate wetting and severe soil drying (AWSD); and (3) continuously flooded (CF). Except for drainage mid-season, the CF regime maintained a continuous flood with 5–10 cm water depth until one week before the final harvest as per recommended farming practices. Soil water potential was monitored at 15–20 cm soil depth with a tensiometer consisting of a sensor of 5 cm length. One tensiometer was installed in each plot of AWMD and AWSD regimes, and readings were recorded at 1200 h each day. When soil water potential reached the threshold of −10 and −15 kPa for AWMD and AWSD regimes respectively, a flood with 5–10 cm water depth was applied to the plots. The amount of irrigation water was monitored with a flow meter (LXSG-50 Flow meter, Shanghai Water Meter Manufacturing Factory, Shanghai, China) installed in the irrigation pipelines. Both irrigation and drainage systems were built between blocks. Each plot was irrigated or drained independently.

Nitrogen application treatments consisted of three N rates including 55, 110, and 165 kg ha^-1^, and representing low amount (LN), normal amount (NN), and high amount (HN) of N, respectively. Nitrogen as urea was applied at seeding phase, early tillering and at panicle initiation (the first appearance of differentiated apex). The proportion of nitrogen application was split into 30%, 40% and 40% respectively, for each of the three phenological phases (vegetative, reproductive and ripening).

### 2.4 Field measurements

Within each sub-plot, five 1 m^2^ quadrats were established for LAI measurements and the collection of field reflectance spectra. On each of the seven sampling dates (Table 1) LAI was measured within each quadrat using an LAI-2200 Plant Canopy Analyzer (LI-COR, Lincoln, NE, USA). A 45° view gap was used to avoid direct sunlight within the sensor and minimize the effects of the illumination and background conditions (Stroppiana et al., 2006). On each occasion, one above-canopy and four below-canopy radiation measurements were collected. All measurements were collected either in the early morning or late afternoon to ensure diffuse lighting conditions.

A chlorophyll content meter (atLEAF+, FT Green, Wilmington, DE) was used to non-destructively measure relative leaf level chlorophyll content. Measurements were collected on the same days as LAI. The atLEAF+ sensor is a handheld device which uses a logarithmic ratio between red and NIR light transmission (650, 900 nm; respectively). The red and NIR regions take advantage of the relationship between high absorption by chlorophyll of red radiant energy and high reflectance of near-infrared energy for healthy leaves and plant canopies. Several previous studies have used the atLEAF+ to monitor leaf chlorophyll content in crops (Novichonok et al., 2016; Padilla et al., 2018) and have compared it to the more widely used SPAD-502 m (Konica Minolta, Inc., Tokyo, Japan) for estimating chlorophyll content (Zhu et al., 2012). Although studies have shown more accurate estimations of chlorophyll from SPAD (Novichonok et al., 2016; Padilla et al., 2018), results from Zhu et al. (2012) indicated a strong relationship between leaf chlorophyll (Chl) content, SPAD values and atLEAF values. The chlorophyll data generated were solely used to help parametrize the PROSAIL model

Canopy spectral measurements were collected using an ASD Field Spec spectroradiometer (Analytical Spectral Devices, Inc., Boulder, CO, USA). A fibre optic cable connected to the ASD with an 18° FOV was used to measure spectra from 1 m above the plant canopy at nadir. Measurements of a white spectralon panel (FSF, Edinburgh, United Kingdom) were used to convert spectral measures of radiance to reflectance. Five spectral measurements were collected and averaged for each 1 m^2^ quadrat. All measurements were made on clear, sunny days between 10:00 and 14:00. The spectral data were resampled to ten Sentinel-2 bands using the band spectral response functions available within the ARTMO software (Verrelst et al., 2012b). These data were used as input to the model inversion approach to retrieve corresponding rice LAI values.

LAI, Chl and spectral measurements from each of the five quadrats per sub-plot were subsequently averaged to provide one set of LAI, Chl and spectral values for each sub-plot (n = 27) per sampling date (n = 7) (Table 1).

### 2.5 Sentinel-2 data acquisition and processing

The Sentinel-2 mission comprises of two satellites launched into orbit in 2015 (Sentinel-2A) and 2017 (Sentinel-2B), respectively. The combination of both satellites provides images every five days. Each satellite carries a Multispectral Imager (MSI) with a swath width of 290 km, and provides data in 13 spectral bands spanning from the visible and near infrared region to the shortwave infrared region, including four bands at 10 m, six bands at 20 m and three bands at 60 m spatial resolution. Sentinel-2 incorporates three bands in the red-edge region, centred at 705, 740 nm and 783 nm, respectively. Sentinel-2 MSI images were obtained from the Copernicus Open Access Hub () with dates corresponding with the dates of field measurements. The Sen2Cor Level-2A processor was used to correct Sentinel-2 Level-1C products (digital number image) for atmospheric effects to generate Level-2A surface reflectance products using the SNAP Toolbox. To retain the red-edge region in the atmospherically corrected images, we chose 20 m as the spatial resolution to resample the data to during the atmospheric correction. Details of the spectral bands retained after pre-processing can be found in Table 2.

### 2.6 LAI retrieval

**Fig. 3:**
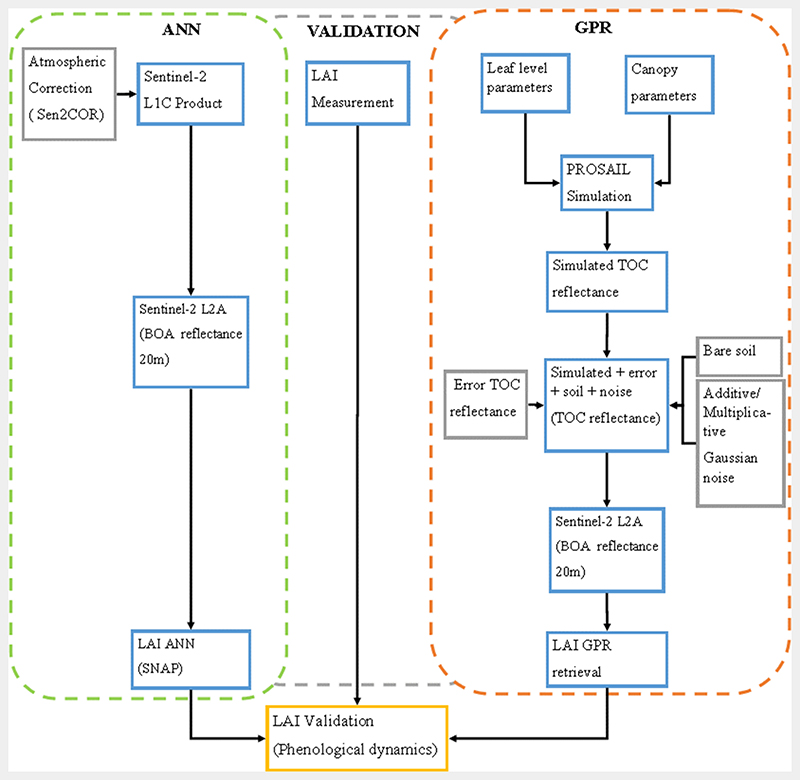
Methodological flowchart divided into ANN and GPR modelling of LAI. Sentinel-2 Level-1C (L 1C) product was corrected atmospherically using Sen2COR in the Snap toolbox to generate Sentinel-2 Level-2A product. Sentinel-2 Level 2A Bottom of Atmosphere (BOA) reflectance was used in generating the LAI ANN product. The leaf level parameters and canopy parameters, using the PROSAIL simulation to generate Top of Canopy (TOC) reflectance. Error Top Of Canopy reflectance, bare soil and additive/multiplicative gaussian noise with the Simulated TOC reflectance using for LAI GPR retrieval.

Two different approaches were used to retrieve LAI: (i) using a hybrid retrieval strategy from the combination of the physical based model (PROSAIL) and the GPR and (ii) using the hybrid ANN model deployable with the SNAP toolbox (Fig. 3).

#### 2.6.1 Radiative transfer modelling using PROSAIL

The PROSAIL model was used to build the database for training the GPR LAI retrieval model. PROSAIL assumes the canopy as a turbid medium for which leaves are randomly distributed. The model (Jacquemoud et al., 2009) refers to the coupling of the PROSPECT leaf optical properties model (Feret et al., 2008) with the SAIL canopy reflectance model (Verhoef, 1984) and has been widely validated and used for LAI estimation (Darvishzadeh et al., 2008; Sehgal et al., 2016; Zhang et al., 2016). PROSPECT-4 simulates leaf reflectance and transmittance for the optical spectrum (400 to 2500 nm) as a function of biochemistry and anatomical structure of the canopy and its leaves. It consists of four-leaf parameters: leaf structure, leaf chlorophyll content, equivalent water thickness and dry matter content (Feret et al., 2008). 4SAIL calculates top-of-canopy reflectance. The 4SAIL input variables are: LAI, leaf angle distribution, the diffuse/direct irradiation ratio, a hotspot parameter and the sun-target-sensor geometry. We opted for Prospect-4 instead of Prospect-D to mirror the PROSAIL model by Weiss and Baret (2016) used in developing S2 Tool box Level-2 product. In doing so, we show the superiority of the GPR model over the ANN model for monitoring the phenological dynamics of LAI particularly during the reproductive and ripening phases of rice.

A LUT was generated using the PROSAIL model to retrieve LAI. The LUT was generated for six fixed parameters (Table 3): LAI, Cab, Cm, Cw, ALA and Sun-sensor azimuth angle. Two thousand random combinations of these parameters were generated within pre-defined parameter ranges based on the collected field data. We selected a database of 2000 as justified by Campos-Taberner (2016) primarily because increasing the number of samples had no significant impact on the accuracy of the retrieval model. We tested for 10,000 20,000 and 50,000 and results were very similar. Moreover, the samples indicate that they did not incur in any overfitting issue and highlighting the good representativity of the simulated data.LAI, Cab, Cm and Cw were sampled using a distribution function suggested by Weiss et al. (2000). Cm, Ca, ALA and Sun-sensor azimuth angle were sampled assuming uniform distributions (Verrelst et al., 2015b).

A dataset of 69 distinct wet and dry soil samples, collected using the ASD spectrometer during the field campaign were also included in the PROSAIL simulations (Verrelst et al., 2019b).

The PROSAIL model top of the canopy full spectra (at 1 nm resolution) were subsequently resampled using Sentinel-2 MSI spectral response functions, to the ten bands as used in the Sentinel-2 level 2 products (Table 2).

Retrieval methods based on simulated data are not affected by noise and measurement uncertainty (Liang, 2007), which can introduce additive and multiplicative band dependent (i.e. applied to individual bands) and independent (i.e. applied to all bands) errors (Verger et al., 2011). Consequently, artificial noise was introduced into the PROSAL model LUT to account for some of the band independent uncertainties. Specifically, white Gaussian noise was added to the output spectra, based on the noise model provided in Eq. (1):

(1) $${ℛ}^{*}(\lambda )=ℛ(\lambda )\left(1+\frac{MD((\lambda )+MI}{100}\right)+AD(\lambda )+AI$$

where R(*λ*) and R*(*λ*) are the raw simulated reflectance for band *λ* and the reflectance with uncertainties for band *λ*, respectively. MD and MI are the multiplicative wavelength dependent noise and the multiplicative wavelength independent noise, respectively. AD and AI are the additive wavelength dependent noise and the additive wavelength independent noise, respectively. After some testing of additive and multiplicative noise, a value of 0.01 for AD and AI, and a value of 2% for MD and MI were used for all simulated wavelength ranges. Similar noise levels were successfully used in a recent study to reduce the over-fitting on the MLRA training database (Upreti et al., 2019).

#### 2.6.2 PROSAIL model inversion using Gaussian processes regression

The simulated canopy reflectance data from PROSAIL was subsequently used to train a GPR model by linking the spectral information to canopy LAI.

Gaussian processes regression (Rasmussen and Williams, 2006) is a nonparametric, Bayesian regression approach, and has been successfully used for the retrieval of LAI in rice (Campos-Taberner et al., 2016). GPR is a probabilistic approximation to non-parametric kernel-based regression, where both a predictive mean (point-wise estimates of LAI) and predictive variance (error bars for the LAI predictions) can be derived. GPR offers a relation between the input (e.g., spectral data) × = [x1, … , xB] ∈ R^B^ and the output variable (i.e., LAI) y ∈ R of the form:

(2) $$\hat{y}=f(x)=\sum_{i=1}^{n}a_{i}K_{\theta }\left(x_{i}x\right)+\alpha_{0}$$

where $\{\text{xi}{\}}_{i=1}^{N}$ are the spectra used in the training phase, *α_i_* ∈ R is the weight assigned to each one of them, *α_0_* is the bias in the regression function, and *K_θ_* is a kernel or covariance function (parametrized by a set of hyperparameters θ) that evaluates the similarity between the test spectrum and all N training spectra.

To generate kernel regression models, a kernel function *K_θ_* to infer the hyperparameters θ and model weight *a* is required. Hence, we used the so-called automatic relevance determination (ARD) kernel, as an alternative generalization of the isotropic SE prior:

(3) $$K(x_{i}x_{j})=vexp\left\{-\sum_{b=1}^{B}\frac{X_{i}^{\left(b\right)}-X_{j}^{\left(b\right)}}{2\sigma_{b}^{2}}\right\}+\sigma_{b}^{2}\delta_{ij}$$

where *ν* is a scaling factor, B is the number of bands, and *σ_b_* is a dedicated parameter controlling the spread of the relations for each particular spectral band *b*. Model hyperparameters are collectively grouped in h = [m, sn, s1, … ,sB], and model weights *a_i_*can be automatically optimized by maximizing the marginal likelihood in the training set (Rasmussen and Williams, 2006; Verrelst et al., 2012a). GPR also provides information about relevance of bands (a ranking of relevant bands), which can be used for identifying the sensitive spectral regions (Campos-Taberner et al., 2016; Verrelst et al., 2016b).

#### 2.6.3 Sentinel-2 application Platform for leaf area index

Among other modules, the SNAP toolbox contains a vegetation processor module that is designed for the retrieval of LAI, canopy chlorophyll content (CCC), canopy water content, fraction of photosynthetically active radiation absorbed by the green elements of the canopy, and fraction of vegetation cover (Weiss and Baret, 2016). The principles governing the retrieval of LAI are based on the hybrid model of PROSAIL adopted for this study and the ANN models adopted as the non-parametric model for model inversion (Weiss and Baret, 2016). Based on a pre-trained neural net, at least one pure LAI pixel was retrieved in each of the experimental subplots from each of the seven Sentinel-2 images (Table 1), accounting for the different phenological phases of rice growth.

### 2.7 Model retrieval accuracy

The LUT simulated with model PROSAIL was used to train GPR into LAI retrieval models applicable to Sentinel-2. In order to assess the GPR inversion process, the model was assessed using k-fold cross-validation (k = 10). For each model, the dataset was randomly divided into 10 equal-sized sub-datasets. From these sub-datasets, K-1 sub-datasets are selected as a training dataset and a single sub-dataset is used as a validation dataset for model testing. The cross-validation process is then repeated 10 times, with each of the 10 sub-datasets used as a validation dataset. This way, all data are used for both training and validation, and each single observation was used for validation exactly once (Verrelst et al., 2015b).

For the validation of the LAI predictions against the actual measured LAI in the field, the GPR and ANN models (will be referred to as GPR and ANN henceforth) were applied to the Sentinel-2 imagery to extract the LAI values for both models from the same imagery. To evaluate the performance of both the GPR and ANN (the SNAP Sentinel-2 MSI model) models with in-situ data, the coefficient of determination (R^2^), the root mean squared error (RMSE) were used in assessing the accuracy of the models (Fig. 3). To determine model accuracy when monitoring the phenological dynamics of both the GPR and ANN models based on different crop management scenarios, both the GPR and ANN LAI models were compared with corresponding field observation plots of LAI phenology patterns over the growing phases of rice.

## 3 Results

### 3.1 Temporal patterns of field measured LAI in response to nitrogen and irrigation treatments

**Fig. 4:**
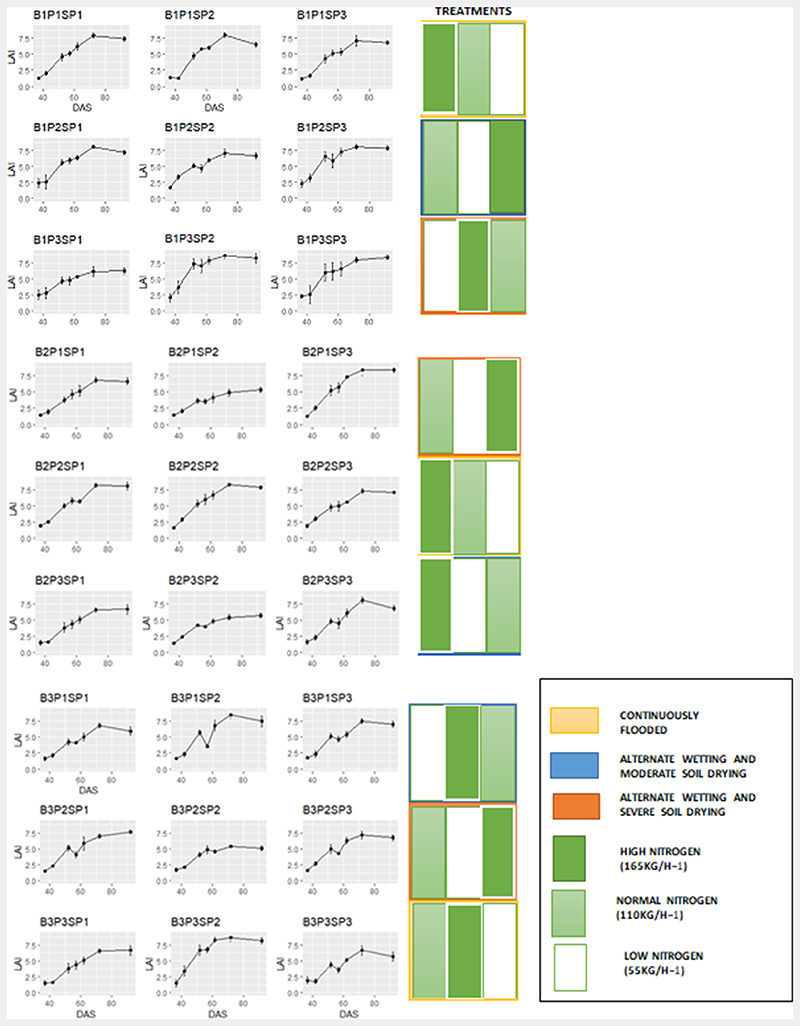
LAI phenology profile for each experimental subplot with errors bars. B represents the block in which each plot is represented. P represents the Plot in which all the sub-plots are represented. SP represents the individual subplots found in each plot. DAS represents Days After Sowing. In total, there are 27 subplots.

LAI values varied with nitrogen and water application rates with the highest LAI values (8.67) observed in plots with HN and CF treatment and lowest values occurring in plots with LN and AWSD treatment (1.17). Within field variability was generally low, with the exception of one subplot (B1P3SP3; see Fig. 2) which showed high variability at the stem elongation and panicle initiation phase (Fig. 4).

Plots that were continuously flooded (CF) showed increasing LAI values across the different phenological phases despite the variation in nitrogen application within the subplots. However, LAI values within AWMD and AWSD plots declined when soil water levels were low (Fig. 3), likely due to the effects of water stress on plant development. These reductions in LAI were observed particularly during the stem elongation phase (see Table 1). In 11 out of the 18 subplots where irrigation applications were altered, declines in LAI values occurred during the vegetative phase. For example, at 57 Days after Sowing (DAS) showed a decline in LAI values for plots with alternating water applications as irrigated water was allowed to drop to −10 and −15 kPa for the AWMD and AWSD plots respectively.

When considering the nitrogen application to each subplot (Fig. 4), it was observed that nitrogen application was an important determinant of LAI dynamics over space and time (Table 1). For instance, B2P1SP2 is characterised by low nitrogen application and AWSD irrigation regime while B2P1SP3 is characterised by high nitrogen application and AWSD irrigation regime. The LAI dynamics show higher LAI during the reproductive and ripening phases with high nitrogen application as compared to low nitrogen application. Similar results were observed when looking at B2P3SP2, which is characterised by low nitrogen application and AWMD irrigation treatment to B2P3SP3 subplot, characterised by normal nitrogen application and AWMD irrigation treatment. Nitrogen played a significant effect in the LAI profiles on both plots with significantly high LAI during the reproductive phase in the normal nitrogen subplots compared to low nitrogen subplot. However, each plot irrespective of nitrogen and irrigation treatment peaked during the reproductive or ripening phenological phase of irrigated rice growth.

### 3.2 PROSAIL-GPR LAI retrieval model validation

The hybrid GPR model inversion training performance was evaluated against the simulated data. The hybrid GPR model explained 65% of variance in LAI (RMSE = 1.21).

**Fig. 5:**
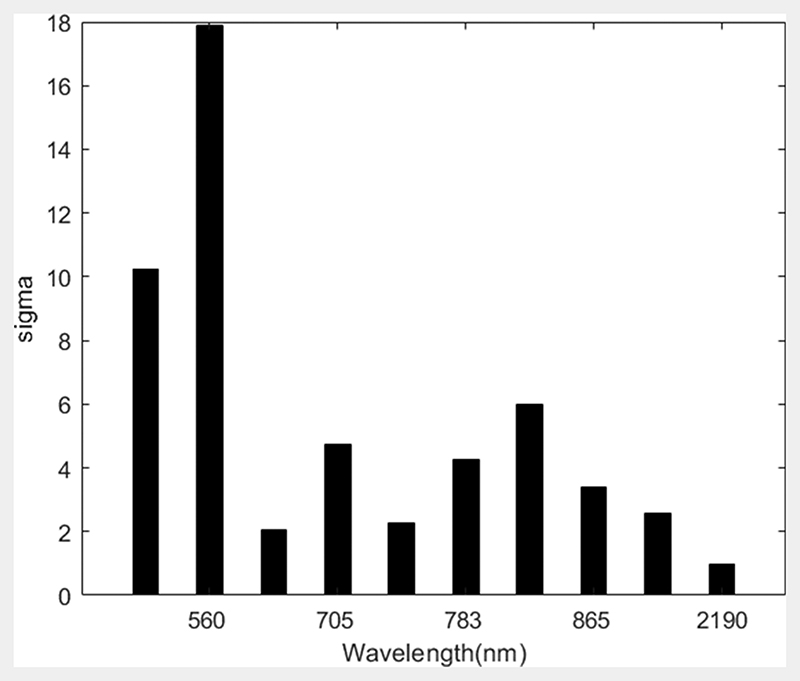
Relevance band histograms for Sentinel-2 simulated bands using the GPR model. The lower the sigma the more important the band.

The red-edge, near-infrared and short-wave infrared bands were the most significant in model development, whilst the blue and green bands in the visible portion of the electromagnetic spectrum contributed least to the model (Fig. 5).

### 3.3 Validation of satellite-derived LAI

**Fig. 6:**
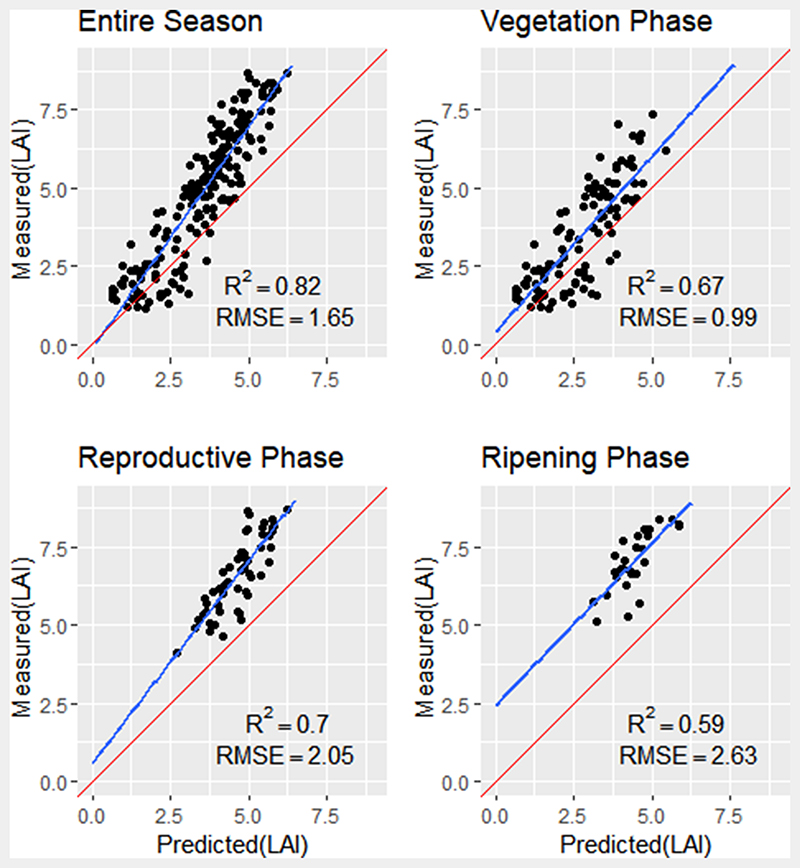
Measured versus predicted LAI from GPR PROSAIL for the entire season (n = 189), the vegetative phase (n = 108), the reproductive phase (n = 54) and ripening phase (n = 27). Diagonal red line represents a 1:1 relationship; blue line is the linear fit between measured and predicted values.

When considering the performance of the hybrid models for predicting LAI against in situ data, the GPR model explained 82% of LAI variation in the model with an RMSE of 1.65 for the entire season. The regression line deviated from the 1:1 line as LAI values increased, leading to underestimation at high LAI values (Fig. 6). When validating LAI at the vegetative and reproductive phases, similar trends were identified, with regression lines deviating from the 1:1 line with increasing LAI (Fig. 6). For the ripening phase, predicted LAI values were underestimated compared to actual field observation (RMSE = 2.63, R^2^ = 0.59, *P* < 0.05; Fig. 5).

**Fig. 7:**
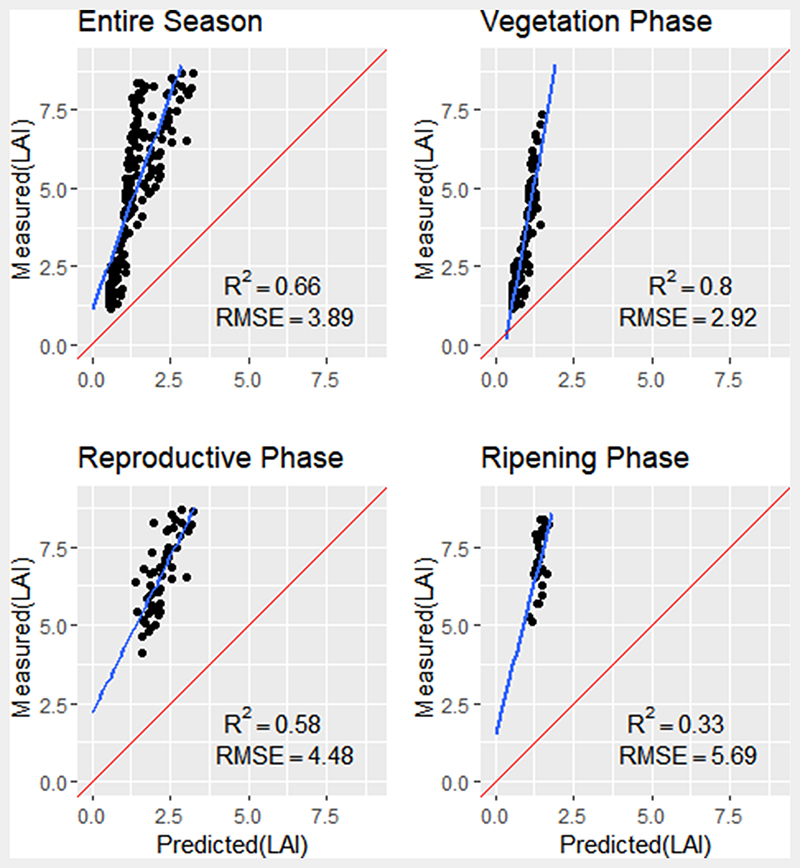
Measured versus predicted LAI from ANN model for the entire season (n = 189), the vegetative phase (n = 108), the reproductive phase (n = 54) and ripening phase (n = 27). Diagonal red line represents a 1:1 relationship; blue line is the linear fit between measured and predicted values.

When comparing the predicted LAI values obtained using the ANN model with in-situ LAI measurements, the ANN model explained 66% of variation in LAI values, albeit under-estimating LAI during of entire phenological phases, leading (RMSE = 3.89, P < 0.05). Similar underestimation trends were found during the reproductive and ripening phases explaining 58% and 33% of the variation in LAI respectively (Fig. 7).

### 3.4 Temporal profile of LAI across altered irrigated and nitrogen regimes

**Fig. 8:**
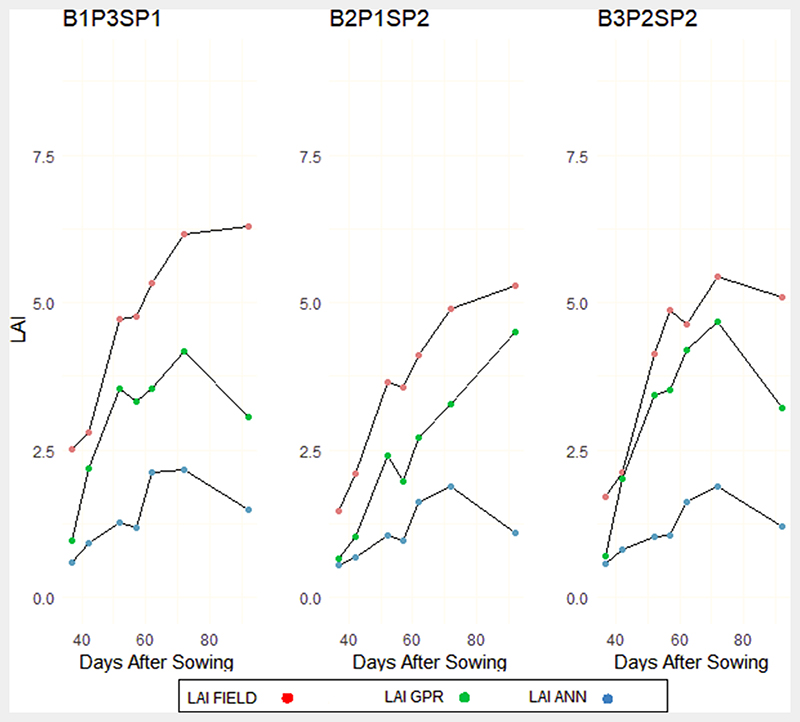
Experimental subplots characterised by Low Nitrogen and Alternative Wetting and Severe Drying (AWSD) regimes for field LAI and hybrid GPR and ANN models.

From the analysis of Low Nitrogen (LN) and Alternative Wetting and Severe Drying (AWSD) subplots, GPR and ANN showed similar profiles during the vegetative phase, however, a general under estimation was identified in both models. The transition from the vegetative to the reproductive phase showed a rapid increase of in-situ LAI. The GPR model showed much higher LAI profile transition, with a decline in LAI values observed in some plots due to the alternative wetting and drying approach adopted. However, there was still underestimation of LAI compared to in-situ measurements. On the other hand, LAI values from the ANN model were consistently low, with peak LAI below 2.4. These peak values are usually attributed to the reproductive phase, showing a high discrepancy between actual ANN LAI phenological with measured LAI in Fig. 8.

**Fig. 9:**
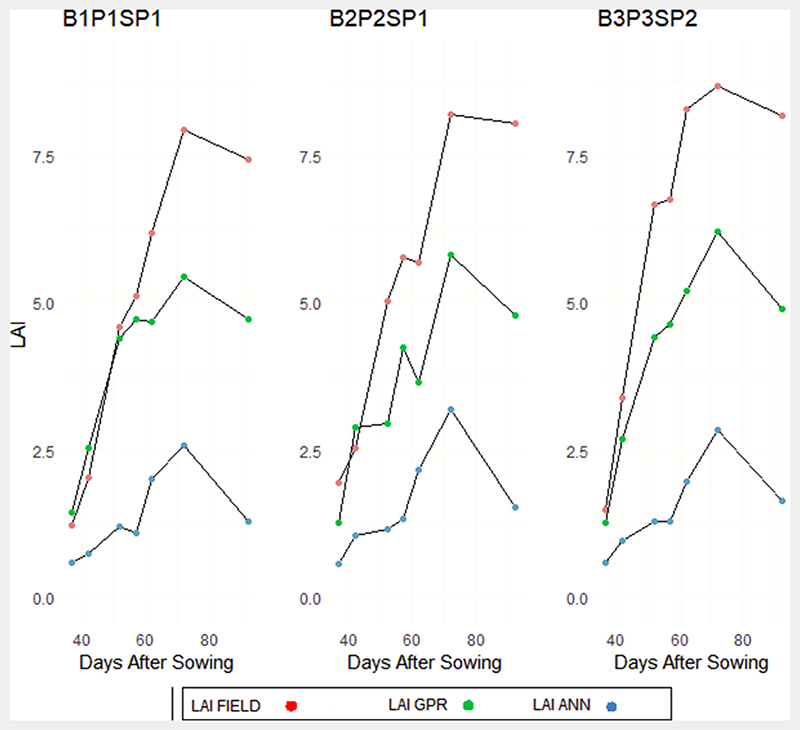
Experimental subplots characterised by High Nitrogen and Continuous Flooding (CF) regimes for field LAI and hybrid GPR and ANN models.

The subplots with High Nitrogen (HN) and Continuous Flooding (CF) showed similar results for the GPR model compared to in-situ measurements (Fig. 9). From the tillering phase (40 DAS), underestimation of LAI values was more evident from the ANN model compared to the measured LAI and GPR estimates. Transitioning to the reproductive phase showed a sharp rise in the LAI profile of subplots with the same nitrogen and water treatments. The GPR results exceeded LAI values of 6, although a general underestimation was the general pattern from the predictive model. In terms of the ANN model, the underestimation was more obvious from the ANN model for the three subplots during the reproductive phase. For the ripening phase, a general decline in LAI was identified compared to in situ measurements for the two hybrid models, however, the pattern of underestimation was more evident with the ANN model (Fig. 9).

**Fig. 10:**
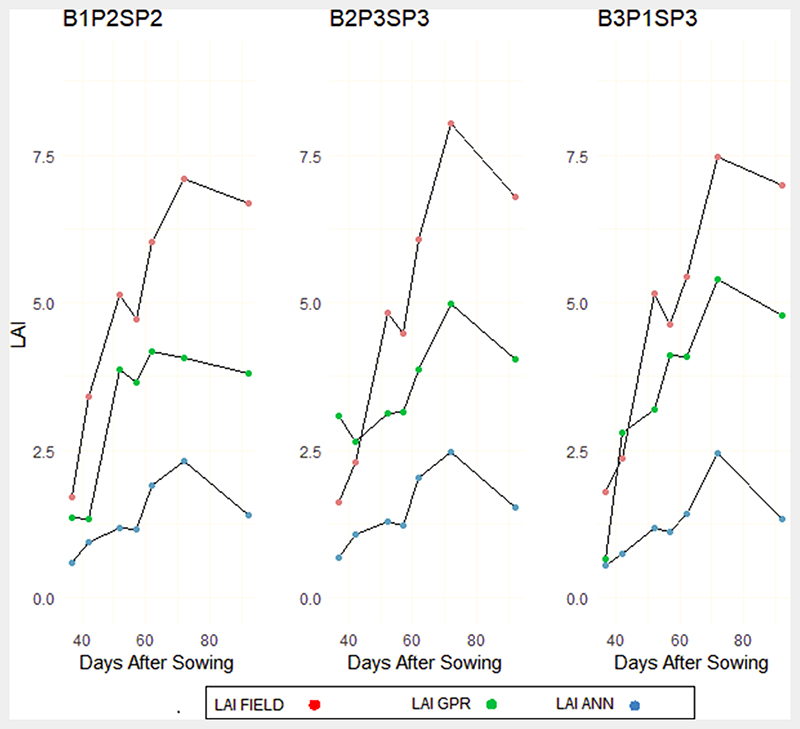
Experimental subplots characterised by Normal Nitrogen and Alternative Wetting and Moderate Drying (AWMD) regimes for field LAI and hybrid GPR and ANN models.

We assessed plots with normal nitrogen (NN) distribution and Alternate Wetting and Moderate Drying (AWMD) plots (Fig. 10). These plots were chosen because there may be a tendency in real scenarios for farm plots to have reduced water supply due to inadequate irrigation systems or drop in water levels at storage point when growing rice. The same patterns identified in Figs. 8 and 9 were reflected in this category, except for the GPR model in the vegetative phase (Fig. 10). For subplots B2P23SP3 and B3P1SP3, the GPR model showed overestimation in one of the plots and underestimation of LAI in the other two plots early in the vegetative phases (Fig. 10). Nevertheless, underestimation was also identified in Fig. 9 during the reproductive and ripening phases. The ANN model estimation of LAI was consistently low as identified in the other phases (Fig. 10).

In summary, ANN and GPR models generally show the same phenological profiles compared to in-situ data, however, the underestimation in ANN models was more significant when estimating the phenology patterns for different nitrogen and irrigation regimes. The same limitations were identified with the GPR model, although the GPR model estimations of rice LAI phenology showed higher variations in LAI results similar to in-situ measurements.

## 4 Discussion

LAI has been identified to have a strong relationship with yield, leading studies to investigate and estimate LAI in order to understand yield trends and patterns (Fang et al., 2014; Gilardelli et al., 2019). With the launch of Sentinel-2, acquiring high spatial, spectral and temporal resolution images as key growth phases of rice has become possible. This study focused on a hybrid retrieval approach by combining the machine learning regression model GPR with PROSAIL simulations for estimating the phenology dynamics of rice LAI over altered irrigation and nitrogen regimes. Furthermore, we assessed the retrieval performance generated by SNAP, which consists of Artificial Neutral Network (ANN) trained by PROSAIL simulations, to understand the seasonal dynamics of rice LAI.

The GPR model showed a positive relationship (R^2^ = 0.65) between the model built from Sentinel-2 data based on the spectral bands and angular configuration in terms of coefficient of determination and RMSE. The relationship of the GPR model at high LAI may have taken into account the addition of soil spectra and noise for optimization of model performance. Similar strategies have been adopted to improve the retrieval estimates of LAI (Campos-Taberner et al., 2016). However, limited variation in soil spectral led to overestimation of LAI when LAI values are low, which aligns with results suggested by Verrelst et al. (2015) as obtaining insufficient soil spectra variation from the experimental area would be limiting. One approach to be considered in future should be to capture a larger variation in soil types, moisture content, the geometric configuration, as well as the roughness of the soil (Jacquemoud et al., 1992). From the trained GPR model, it was possible to identify the most significant spectral bands for LAI retrieval. The bands along the red edge, near-infrared and short-wave infrared, were more important compared to the blue and green bands along the visible part of the electromagnetic spectrum as shown in Fig. 4. The results observed were in agreement with earlier observations (Darvishzadeh et al., 2008; Delegido et al., 2011; Verrelst et al., 2015b).

When validating the GPR and ANN models against in-situ LAI measurements, the GPR PROSAIL model exhibited a better agreement with in-situ measurements compared to ANN across the entire growing season (Figs. 6 and 7). The improvement may be greater because of the transparent nature of the GPR model, which allows the use of simple to complex kernel functions for parameterisation of the model, while also providing uncertainty estimates of the mean value of prediction (Upreti et al., 2019). Further investigation into the key phenology phases saw improved estimation accuracy from the GPR model as compared to ANN PROSAIL during the vegetative, reproductive and ripening phases. In terms of the ANN model accuracy, the RMSE may present a bias due to trends in time series data. The ANN model displayed consistently low LAI estimates through all growing phases, leading to high model bias between estimated and predicted values of the entire season and particularly during the reproductive and ripening phases (Figs. 9 and 10), with underestimation of LAI apparent at each phenology phase.

Operational LAI products (Sentinel-2 and MODIS) have been identified to provide underestimated LAI in different seasons (Xie et al., 2019). This was evident when assessing the phenological patterns of rice LAI in this study. LAI values remained below 4 in plots with increased irrigation and nitrogen application. PROSAIL models have shown to underestimate LAI in dense vegetation (Verrelst et al., 2015b), even though they have shown to be compensated when inverted with machine learning algorithms (Campos-Taberner et al., 2016). Although GPR and ANN models underestimated LAI, especially during the reproductive and ripening phases as shown in Figs. 8, 9 and 10, the GPR phenology patterns were closely related to in-situ measurements, with some plots showing overestimation during the vegetative phase (Fig. 9).

During the vegetative phase, LAI of rice farms with adequate water supply show rapid leaf production as a result of elevated carbon dioxide concentration (Grashoff et al., 1995). The increased leaf production was identified in CF plots with high and normal nitrogen applications in Fig. 4. This applies particularly for indeterminate growing species and under nonlimiting supply of nutrients. This was also evident in plots where water supplies were withheld for a couple of days during the vegetative phase despite a dip in LAI values. Although the GPR and ANN models identified similar field observation LAI patterns, underestimation was evident during the vegetative phase. During the reproductive phase, LAI at heading increased with increasing nitrogen rate, which also coincides with the peak of LAI values (Sharma and Yadav, 1999). Fig. 4 shows similar results with rice LAI peaking in most of the subplots and the rise of LAI curve as a result of the nitrogen application. The GPR model accounted for an increase in LAI values up to 6. On the other hand, the ANN model did not exceed LAI values of 4 during the heading phases. Previous studies assessment of the performance of the ANN model have compared model performance when LAI values did not exceed 4 (Pasqualotto et al., 2019; Xie et al., 2019), although in other studies ANN LAI estimates exceeded 4 (Vanino et al., 2018; Xie et al., 2019). Therefore, further validation of the models is needed in other regions. The ripening phase ushered in a sharp decline of LAI due to translocation of accumulated plant reserves to the panicle (Sharma and Yadav, 1999). This was evident in the in-situ LAI measurements and the GPR and ANN models. However, underestimation was observed in the GPR model with higher underestimation found in ANN LAI. The underestimation results obtained from both models may have been as a result of changes in spectral reflectance over a relatively small portion of the experimental subplots (experimental sub-plot > 0.5 ha) leading to anisotropy effects of reflectance based on the spatial resolution of Sentinel-2. Furthermore, ANNs are black box in nature, and can be unpredictable if training and validation data deviate from each other even slightly (Verrelst et al., 2015b). Whereas, GPR provides insights in bands carrying relevant information and also in theoretical uncertainty estimates, thus partially overcoming the black box problem.

Despite the superior performance of GPR for estimating LAI, GPR is computationally expensive if trained on large sets of simulations (Upreti et al., 2019; Verrelst et al., 2016a) and will not necessarily alleviate the limitations of RTMs, such as the ill-posed inverse problem or the constrained model’s capability of reproducing the measured (canopy bidirectional) spectral signals (Berger et al., 2018). Yet, the major benefit of GPR entails providing a comprehensive training data base for the machine learning regression model without the necessity of in-situ data collection (although this is still required for validation). Furthermore, the LUT can be modified based on the specific application by implementing existing knowledge and concepts of experienced users.

Finally, to combat spectral reflectance issues due to experimental plot sizes, future studies should investigate the retrieval of GPR and ANN models over different phenology phases to understand LAI dynamics in a bid to improve global LAI estimation. Furthermore, developing models for specific regions should be investigated in future studies.

## 5 Conclusion

This study focused on determining the potential of PROSAIL and Gaussian Processes Regression (GPR) for estimating the phenological dynamics of irrigated rice LAI from Sentinel-2 data. Subsequently, we compared the performance of hybrid GPR and hybrid ANN model generated from Sentinel-2 Application Platform (SNAP) for estimating the seasonal LAI dynamics of rice fields with altered nitrogen and water applications at different phases of crop growth.

The GPR model outperformed the ANN model in LAI estimation during the reproductive and ripening phases while at the same time offering uncertainty estimates. Further, in the analysis of both models, the ANN model showed underestimation of LAI, particularly in the reproductive and ripening phases of LAI development while the GPR model showed some overestimation during the vegetative phases. When estimating the phenological dynamics of LAI, the LAI growth curve was much closer to in-situ measurements when using GPR compared to ANN during the reproductive and ripening phases, with less underestimation. Results suggest that the GPR model more accurately estimate the phenological dynamics of rice in altered management practices. The study opens opportunities for further studies in other crop types, regions and growing seasons in other to validate and improve global LAI estimation.

## Figures and Tables

**Table 1:** Field measurements and data used for calibration and verification of the retrieval scheme. The Sentinel-2 data were acquired on the same day field measurements were conducted. Spec data – Spectral data; LAI – Leaf Area Index; Chl – Chlorophyll.

Phenology	Growth phase	Date	Days After Sowing (DAS)	Spec data	LAI	Chl	Sentinel-2
Vegetative	Early Tillering	30-01-18	37	✓	✓	✓	✓
	Tillering	04-02-18	42	✓	✓	✓	✓
	Stem Elongation	14-02-18	52	✓	✓	✓	✓
	Stem Elongation	19-02-18	57	✓	✓	✓	✓
Reproductive	Panicle Initiation	24-02-18	62	✓	✓	✓	✓
	Heading	06-03-18	72	✓	✓	✓	✓
Ripening	Milk	16-03-18	92	✓	✓	✓	✓

**Table 2:** Sentinel-2 Multispectral Instrument (MSI) spectral bands (B) retained after pre-processing.

Sentinel-2 Bands	B2	B3	B4	B5	B6	B7	B8	B8A	B11	B12
Central Wavelength (μm)	0.49	0.56	0.67	0.71	0.74	0.783	0.84	0.87	1.61	2.19
Resolution (m)	10	10	10	20	20	20	10	20	20	20
Bandwidth (nm)	65	35	30	15	15	20	115	20	90	180

**Table 3:** Range and distribution of input parameters used to establish the synthetic canopy reflectance database for use in the LUT.

Model parameters	Range	Mean/standard deviation
Leaf parameters: PROSPECT-4			
N	Leaf structure index	1.2–2.5	
LCC	Leaf Chlorophyll Content	10.0–55	35/20
Cm	Leaf dry matter content	0–0.03	
Cw	Leaf water content	0–0.05	
Canopy variables: 4SAIL			
LAI	Leaf area index	0.2–9	5.5/4
soil	Soil scaling factor	0–1	
ALA	Average leaf Angle	40–80	
HotS	Hot spot parameter	nil	
